# Insights into the control of taxane metabolism: Molecular, cellular, and metabolic changes induced by elicitation in *Taxus baccata* cell suspensions

**DOI:** 10.3389/fpls.2022.942433

**Published:** 2022-07-29

**Authors:** Edgar Perez-Matas, Abdulsamie Hanano, Elisabeth Moyano, Mercedes Bonfill, Rosa M. Cusido, Javier Palazon

**Affiliations:** ^1^Secció de Fisiologia Vegetal, Facultat de Farmacia i Ciències de l’Alimentació, Universitat de Barcelona, Barcelona, Spain; ^2^Department of Molecular Biology and Biotechnology, Atomic Energy Commission of Syria, Damascus, Syria; ^3^Departament de Ciències Experimentals i de la Salut, Universitat Pompeu Fabra, Barcelona, Spain

**Keywords:** *Taxus baccata*, cell cultures, paclitaxel, coronatine, gene expression, lipid droplets, taxane accumulation

## Abstract

More knowledge is needed about the molecular/cellular control of paclitaxel (PTX) production in *Taxus* spp. cell cultures. In this study, the yield of this anticancer agent in *Taxus baccata* cell suspensions was improved 11-fold after elicitation with coronatine (COR) compared to the untreated cells, and 18-fold when co-supplemented with methyl-β-cyclodextrins (β-CDs). In the dual treatment, the release of taxanes from the producer cells was greatly enhanced, with 81.6% of the total taxane content being found in the medium at the end of the experiment. The experimental conditions that caused the highest PTX production also induced its maximum excretion, and increased the expression of taxane biosynthetic genes, especially the flux-limiting BAPT and DBTNBT. The application of COR, which activates PTX biosynthesis, together with β - CDs, which form inclusion complexes with PTX and related taxanes, is evidently an efficient strategy for enhancing PTX production and release to the culture medium. Due to the recently described role of lipid droplets (LDs) in the trafficking and accumulation of hydrophobic taxanes in *Taxus* spp. cell cultures, the structure, number and taxane storage capacity of these organelles was also studied. In elicited cultures, the number of LDs increased and they mainly accumulated taxanes with a side chain, especially PTX. Thus, PTX constituted up to 50-70% of the total taxanes found in LDs throughout the experiment in the COR + β - CD-treated cultures. These results confirm that LDs can store taxanes and distribute them inside and outside cells.

## Introduction

Taxol^®^, known generically as paclitaxel (PTX), is one of the most effective anti-cancer drugs in current use. The Food and Drug Administration (FDA) approved PTX for the treatment of refractory metastatic ovarian cancer in 1992, metastatic breast cancer (refractory or insensitive to anthracyclines) in 1994, and subsequently, non-small cell lung cancer and AIDS-related Kaposi’s sarcoma. PTX is also currently administrated against prostate, breast, ovarian, stomach, cervical, esophageal, testicular, and pancreatic cancers, as well as for AIDS-related Kaposi’s sarcoma, and leukopenia^1^. Additionally, PTX is now under study for the treatment of neurodegenerative conditions such as Alzheimer’s or Parkinson’s disease (Zhang et al., 2005) and other health problems related with the stabilization of microtubule-associated proteins, such as psoriasis (Ehrlich et al., 2004). Consequently, PTX is perhaps the most important antitumor agent in history. The global PTX market was valued at US$ 4.51 billion in 2021 and is expected to reach over US$ 11.16 billion by 2030, with a compound annual growth rate (CAGR) of 12.5% during the forecast period 2022 to 2030 (Sofias et al., 2017). More than 20 companies are involved in the development and commercialization of new pharmaceutical forms of PTX in several countries, especially the United States, which accounts for almost 40% of the global oncology market (Sofias et al., 2017).

Paclitaxel is a poorly soluble diterpene pseudoalkaloid with a very complex chemical structure. It was initially extracted from the inner bark of *T. brevifolia* in the order of 0.01% referred to dry weight, and this ratio is seriously fluctuating with the seasons (Cameron and Smith, 2008). Later, PTX is found in other *Taxus* species, also in very low quantities. According to Bedi et al. (1996), a single course of treatment requires between 2.5 and 3 g of PTX, which implies harvesting the inner bark of about eight 60-year-old trees. Consequently, PTX extraction from *Taxus* trees is not ecologically sustainable. Although the chemical synthesis of PTX was achieved in 1994 (Holton et al., 1994a,b; Nicolaou et al., 1994), this approach remains commercially unfeasible. Alternatively, PTX can also be obtained by semi-synthesis, starting from more abundant taxanes such as 10-deacetylbaccatin III and baccatin III, which can be extracted from yew needles, a natural and renewable source (Guénard et al., 1993). The semi-synthesized compound, known as docetaxel or Taxotere^®^, is more soluble in water than PTX and 2.7 times more effective against certain cancers (Amato, 1992), and is widely applied in anti-cancer therapy. Nevertheless, the biotechnological production of PTX by means of optimized *Taxus* spp. cell cultures is the current method of choice, as the cells suspension can be grown under controlled and cost-effective conditions. For example, Plant Cell Fermentation (PCF^®^) technology has been developed by Phyton Biotech (United States and Europe), the world’s largest supplier of PTX.

Although its effectiveness as anti-cancer drug, PTX has several adverse effects when utilized with the formulation vehicles Cremophor EL (CrEL), absolute ethanol, or similar alternatives. These products trigger severe anaphylactoid hypersensitivity reactions, hyperlipidemia, abnormal lipoprotein patterns, the aggregation of erythrocytes, and peripheral neuropathy (Gallego-Jara et al., 2020). Therefore, considerable research has been performed to developing alternative delivery systems for PTX. For example, nanocarriers that can specifically target cancer cells. The first PTX nano-formulation was Abraxane (ABX) that contains albumin nanoparticles allowing a higher PTX dose to be administered with far fewer side effects. Subsequently, other nanomaterials have been used, such as polymeric lipidic nanoparticles (e.g., PICN^®^), polymeric micelles (e.g., Cynviloq^®^, Nanoxel^®^, and Paclical^®^), and liposomes (e.g., Lipusu^®^), and many others are in clinical trial phases (Sofias et al., 2017). The high specificity of nanocarriers is achieved by surface functionalization with monoclonal antibodies that have affinity for the target antigen (targeted nanomedicines) (Nevala et al., 2017). Juan et al. (2020) reported that antibody-conjugated nanoparticles loaded with PTX were highly effective in breast cancer therapy, allowing controlled release and accumulation of the therapeutic agent in the tumor sites.

Drug delivery systems have also been improved by the incorporation of cyclodextrins (CDs), which by hosting lipophilic molecules can enhance their permeability and absorption. After treatment Caco-2 cells with an inclusion complex consisting of rhodamine-labeled random methyl-β-CDs and a fluorescent PTX derivative, Haimhoffer et al. (2019) detected both components in intracellular vesicles. The loading capacity and encapsulation efficiency of nanocarriers in cancer therapy can also be increased by conjugation with CDs.

A new efficient nanocarrier system for drugs and other products with potential application in nanomedicine is based on lipid droplets (LDs). Formed in the endoplasmic reticulum, LDs are small organelles present in animal, plant, fungi and possibly certain lipophilic prokaryotics (Murphy, 2012; Hanano et al., 2015; Hanano et al., 2018). LDs have a core of neutral lipids (usually triacylglycerols and steryl/wax esters) and enclosed by a monolayer of phospholipids into which certain classes of proteins are integrated, generally with structural and/or metabolic roles (Huang, 2018). LD size ranges from 40 nm to 1 mm or more, reaching 100 mm in adipocytes (Zhao et al., 2022). However, LD size, location, and their lipid and protein content can be varied by environmental stimuli and the cellular state (For Review: Huang, 2018). Initially, LDs have been described as reservoirs of high-energy metabolites and other constituents (Huang, 2018). Nowadays LDs are known to be effectively involved in stress response, pathogen resistance, lipid-based signaling, and many other processes (van der Schoot et al., 2011; Chapman et al., 2012; reviewed by Huang, 2018).

In the last decade, research has revealed the high potential of LDs for application in molecular pharming and bioremediation, as well as in nanomedicine to deliver lipophilic anticancer drugs. As lipid-rich low-density particles, LDs can be easily isolated by flotation/centrifugation, rendering them suitable for diverse biotechnological applications. Bhatla et al. (2010) showed the usefulness of LDs carrying oleosins fused with a recombinant protein essential for human health (e.g., insulin, hirudin, or growth factors) obtained from oil seeds of transgenic plants. Purification of the LDs and cleavage of the fusion protein constitutes a simple method for the large-scale preparation of the purified target protein.

A promising application of these organelles in environmental remediation was reported by Hanano et al. (2016), who observed that dioxins, potent environmental toxins, were taken up and absorbed into the inner oily core of LDs isolated from seeds of date palm (*Phoenix dactylifera* L.). Hanano et al. (2022) also demonstrated that LDs from date palm seeds, *T. media* and *Arabidopsis thaliana* have a high capacity to sequester PTX *in vitro*, as almost 98% of the PTX in an aqueous solution was found partitioned into plants LDs. Zhao et al. (2022) prepared high-purity artificial LDs (aLDs), which can be preserved for a long period by freeze-drying and Liang et al. (2021) reported that cell-derived LDs acted as controllable and biocompatible carriers able to deliver anticancer drugs and significantly inhibit tumor growth *in vivo* with minimal side effects. The isolated LDs maintained their key physiological functions and interacted with other organelles to enhance the effect of cancer photodynamic therapy by encapsulation with a lipid-conjugated photosensitizer (Pyrolipid).

Manipulation of LD properties and functions, such as size, distribution, and intracellular communication, may provide a controllable and universal platform to deliver anticancer payloads. In summary, this organelle-based delivery system could extend cell-based therapy to a subcellular level, providing new perspectives for drug delivery and holding promise for clinical application in the near future.

In the present work, the mechanisms that control the production and secretion of taxane were studied in *T. baccata* cell suspensions after treatment with the elicitor coronatine (COR, 1 mM) and β-CDs (50 mM) in comparison with control conditions. To gain further insight into taxane metabolism in cell cultures, the role of LDs in the accumulation, partitioning and trafficking of PTX and related taxanes, and the expression pattern of PTX biosynthetic genes during the experiment were analyzed.

## Material and methods

### Plant material

Cell suspension cultures were established from a stable callus line of *T. baccata* derived from sterilized young stems, as previously described by Cusido et al. (2002). After removing the needles, explants were first immersed in 70% (v/v) ethanol for 1 min, then for 15 min in 0.01% (w/v) HgCl_2_ and finally 30 min in 1.5% (v/v) sodium hypochlorite supplemented with 10 drops of Tween 20. After each treatment, explants were rinsed three times with sterile distilled water and finally placed with the inner cut surface in contact with the induction medium, consisting of Gamborg’s B5 medium (Gamborg et al., 1968) supplemented with B5 vitamins, 3% sucrose and the growth regulators (4 mg/L) 2,4-dichlorophenoxyacetic acid, (1 mg/L) kinetin and (0.5 mg/L) gibberellic acid. The pH was adjusted to 5.8 prior to autoclaving. After 3-4 weeks, the different callus tissues formed were separated from the explants and placed together. Calli were grown in solid Gamborg’s B5 medium (Gamborg et al., 1968) supplemented with 2x B5 vitamins, 0.5% sucrose, 0.5% fructose, and the growth regulators: (2 mg/L) 1-naphthaleneacetic acid, (0.1 mg/L) 6-benzylaminopurine and (0.5 mg/L) gibberellic acid. The pH was adjusted to 5.8 prior to autoclaving. The cells were cultured in the indicated growth medium (GM) at 25°C in darkness and subcultured every two weeks to obtain enough friable and vigorous calli to establish cell suspension cultures.

In order to reduce excessive production and release of polyphenols into the medium, an antioxidant solution (Kim et al., 2005) consisting of L-glutamine (14.6 g/L), ascorbic acid (2.5 g/L) and citric acid (2.5 g/L) was added to both the induction medium and GM after autoclaving at a rate of 10 mL/L. The solution was filter-sterilized (0.22 mm sterile PES filters, Millipore, Billerica, MA, United States).

### Cell suspension culture conditions and elicitation

Cell suspensions were cultured using a two-stage system as described by Cusido et al. (2002) and Palazon et al. (2003). After 14 days of cultivating the *T. baccata* cells in liquid GM for biomass production, 3 g of cells were transferred into 10 mL of taxane production medium (PM) in a 175 mL flask capped with a MagentaTM B-cap (Sigma Aldrich, St Louis, MO, United States). The PM, which promoted taxane biosynthesis in detriment of growth, consisted of Gamborg’s B5 liquid medium (Gamborg et al., 1968) supplemented with 3% sucrose and the growth regulators (2 mg/L) picloram, (0.1 mg/L) kinetin, and (0.5 mg/L) gibberellic acid at pH 5.8. The antioxidant solution was also added in the proportion and conditions already described. Cell cultures were kept in the dark at 25 ± 0.2°C and 100 ± 1 rpm in an orbital shaker-incubator (Adolf Kühner, Schweiz).

Taxane production was stimulated by adding elicitors at the beginning of the second phase of culture (Onrubia et al., 2013). Elicitation was performed with 1 mM of coronatine (COR) (Sigma Aldrich, St Louis, MO, United States) and 50 mM of randomly methylated-β-cyclodextrins (β-CDs) (Sigma Aldrich, St Louis, MO, United States), which were added to the cell suspensions separately or together. The elicitors were sterilized by autoclaving (β-CDs) or filter-sterilized (COR).

### Biomass determination and viability assay

Fresh weight was determined by filtering the cells with 80 mm Nylon filters. The cells were freeze-dried to obtain the dry weight and perform the taxane extraction. Cell viability was evaluated as described by Exposito et al. (2010). A small aliquot of each sample was incubated for 5 min in B5 medium containing 0.01% (w/v) propidium iodide (ICN Biomedicals, Costa Mesa, CA, United States) for the selective labeling of dead cells, and 0.01% (w/v) fluorescein diacetate (Sigma Aldrich, St Louis, MO, United States) for the selective labeling of live cells. Fluorescence was observed under a fluorescence microscope (Leica DMIRE2, Leica Microsystems Inc., Wetzlar, Germany) coupled to a camera (Leica DFC360-FX) and using specific filters. Samples were harvested after 0, 8, 12, 16, 20, and 24 days of treatments.

### Isolation of lipid droplets from cell suspensions

Lipid droplets were isolated from *T. baccata* cell suspensions according to Hanano et al. (2016, 2022) with slight variations. Briefly, the whole fresh weight of each sample (between 2.5 and 4 g depending on the elicitation treatment and day of collection) was harvested by filtration with 80 mm Nylon filters and finely pulverized with liquid nitrogen using a mortar and pestle. The powder was immediately hydrated with 5 mL of buffer A (100 mM potassium pyrophosphate, 100 mM sucrose and pH 7.4). The mixture was gently vortexed for 5 min and centrifuged for 10 min at 10,000 g (AvantiTM J-20 XP centrifuge, Beckman Coulter Inc., CA, United States). The supernatant was subjected to a second centrifugation at 100,000 g (OptimaTM L-90K Ultracentrifuge, Beckman Coulter Inc., CA, United States) for 1 h using special polycarbonate centrifuge tubes (Beckman Coulter^®^), and the obtained pellet was freeze-dried and stored for taxane analysis. After the second centrifugation, the upper 3 mL layer consisting of LDs was carefully collected using a glass Pasteur pipette, suspended in 1 mL of buffer B (100 mM potassium pyrophosphate, pH 7.4) and stored at 4°C for further analysis.

### Determination and quantification of lipid droplets

Isolated LDs were analyzed and quantified using a fluorescence microscope. In addition, direct samples were taken from the cell cultures to determine and quantify LDs within cells (LDs/cell). For that purpose, the lipophilic TopFluor^®^ Cholesterol or Bodipy Cholesterol dye [23-(dipyrrometheneboron difluoride)-24-norcholesterol] (Avanti^®^ Polar Lipids, AL, United States) was used for selective labeling of LDs. The dye was first dissolved in dimethyl sulfoxide (DMSO) at a concentration of 1 mg/mL. Then, as in Zhou et al. (2019), 2 or 3 drops (300 mL approx.) of TopFluor^®^ Cholesterol were added to 1 mL of culture medium to obtain a final concentration of 10 mM. The solution was stirred and left for 10 min in the dark at room temperature. Finally, the supernatant was removed and washed with distilled water before observation.

The freshly stained LDs were observed under a fluorescence microscope using a 10X ocular and 10X objective lens (Leica DMIRE2, Leica Microsystems Inc., Wetzlar, Germany) coupled to a camera (Leica DFC360-FX) with red and green fluorescence filters (excitation, 545 and 480 nm; emission, 620 and 535 nm, respectively), exposure of 1.233 s and gain 1.55. Isolated LDs and LDs/cell were quantified using the Java-based image processing program imageJ and were expressed as number of LDs or LDs/cell per area (LDs/cm^2^ and LDs/cell/cm^2^, respectively).

### Total taxane extraction and quantification

Taxanes were extracted from the culture media and lyophilized cells as previously described by Onrubia et al. (2013). Ten mL of media was mixed and vortexed for 2 min with 5 mL of dichloromethane (DCM), followed by 1 h sonication at 25°C. After that, the organic phase was recovered using a glass Pasteur pipette and allowed to evaporate until dryness. For taxane extraction from freeze-dried cells, 8 mL of methanol:water (9:1 v/v) was added to the total amount of lyophilized cells (200 mg approx.), warmed up for 8 min in the microwave at 80 W, and then filtered with 80 mm Nylon filters. The process was repeated twice and both methanolic extracts were mixed. After adding 16 mL of hexane, the samples were centrifuged at 2,500 g (NEYA 8 Bench Top Centrifuge, Remi Group, India) for 20 min at room temperature. The aqueous phase was recovered, mixed with 8 mL of DCM and 4 mL of water, and vortexed until an emulsion of both phases was obtained. After recovering the organic phase, the aqueous phase was vortexed again with 4 mL of DCM. Finally, both organic extracts were mixed and allowed to evaporate. All samples were resuspended in 500 mL of methanol and filtered (0.45 mm PVDF filters, Millipore, Billerica, MA, United States) prior to analysis.

HPLC analyses were performed with a Water Acquity Ultra Performance LC system (Waters, Milford, MA, United States). Taxanes were separated in a Discovery HS F5-5 column 25 cm × 4.6 mm, 5 μm (Supelco, Bellefonte, PA, United States) using a mobile phase composed of acetonitrile (A) and water (B) with the following gradient (min/A%): 0/25, 38/60, 40/100, 45/25, and 55/25, with a flow rate of 1 mL/min and injection volume of 10 mL. Identification criteria included retention time, UV spectra, and co-chromatography, with the standard peak homogeneity determined by a photodiode array detector when spiked with an authentic standard. Taxanes were quantified by integrating the corresponding peak in the standard calibration curve of each target compound: 10-deacetylbaccatin III (DABIII), baccatin III (BACIII), 10-deacetyltaxol (DAT), cephalomannine (CEPH) and paclitaxel (PTX). All standards were provided by Abcam (Cambridge, United Kingdom).

### Transcripts analysis

Total RNA was isolated from 200 mg of frozen cells at 0, 6, 12, 24, 48 h using the Real Plant RNA Kit (REAL, Valencia, España) according to the manufacturer’s instructions. RNA concentration of each sample was determined using a NanoDrop ND-1000 spectrophotometer (NanoDrop Technologies Wilmington, DE, United States). cDNA was prepared from 1 μg of RNA with SuperScript IV Reverse Transcriptase (Invitrogen, CA, United States). The expression level of the *TXS*, *T7βOH*, *BAPT*, *DBTNBT* and *ABC* genes was determined by qRT-PCR using SYBR Green Mastermix (Biorad, Hercules, CA, United States) in a 384-well platform system (LightCycler^®^ 480 Instrument, Roche, United States). Gene-specific primer sequences were obtained from previous studies of our research group (Onrubia et al., 2010, 2011; Sabater-Jara et al., 2014) (Supplementary Table 1). Reaction mixture and thermos-cycling program were carried out as described before (Hanano et al., 2022). The amplification efficiency of each primer pair was determined empirically by 1/25 serial dilutions of cDNA and calculated as described by Applied Biosystems^®^ instructions. The *TBC41* gene was selected as a reference to normalize genes expression (Sabater-Jara et al., 2014; Vidal-Limon et al., 2018; Yanfang et al., 2018). Data were analyzed using LightCycler^®^ analysis software v4.1.

### Statistics

Statistical analysis was performed using Excel and RStudio software. All data regarding biomass and viability determination, taxane quantification and transcription profiling were expressed as the average of three independent determinations ± standard deviation (SD). Data concerning determination and quantification of isolated LDs and LDs/cell were obtained using the Java-based image processing program imageJ and were expressed as the average of 10 and 20 independent determinations ± SD, respectively. The multifactorial ANOVA analysis followed by the Tukey multiple comparison tests were used for statistical comparisons. A P-value of <0.05 was assumed for significant differences.

## Results

### Growth and viability of *Taxus baccata* cell cultures

Cell suspensions were maintained for 12 days in the corresponding optimum GM and the biomass obtained was transferred to the optimum PM, which promotes specialized metabolism in detriment of growth. At the beginning of the second stage, cell cultures were supplemented with COR alone (COR) or COR + β - CDs (CC). Samples were harvested every 4 days from 8 to 24 days after elicitation.

The growth curve (Supplementary Figure 1), measured as fresh weight, indicated that the biomass increased in control conditions, even though the cells were maintained in the optimum medium for taxane production. Thus, after a lag phase of 12 days, the biomass actively increased until day 20, when the culture entered the stationary phase. At the end of the culture, the fresh weight was 400 g/L, which corresponded to an increase of 33.3% in relation to the inoculum.

In contrast, when cell cultures were supplemented with the elicitor COR (Supplementary Figure 1), the fresh weight decreased during the first 12 days and slightly increased thereafter. The values suggest that β-CDs did not lessen the negative impact of COR on growth, which did not differ significantly between COR- and CC-treated cultures.

The negative impact of COR on growth was confirmed by measuring the dry weight as shown in Supplementary Figure 2., but this indicated that β-CDs had a counteractive effect, as the highest values corresponded to the CC cells. The fact that both the fresh and dry weight results of COR-treated cultures followed the same pattern suggests that their lower fresh weight was not due to osmotic changes but to a decrease in living cells, which was supported by the viability study (ranging from 87% at day 0 to 45% at day 24 in COR-treated samples, from 87 to 55% in CC-treated cultures and from 87 to 65% in control conditions).

### Structure and quantification of lipid droplets

Lipid droplets are organelles with important cellular functions that originate in the cytosol-facing domain of the endoplasmic reticulum (Hanano et al., 2022). In the present study, the morphology and abundance of LDs were determined in *T. baccata* cell cultures maintained in control conditions or supplemented with COR or CC.

**FIGURE 1:**
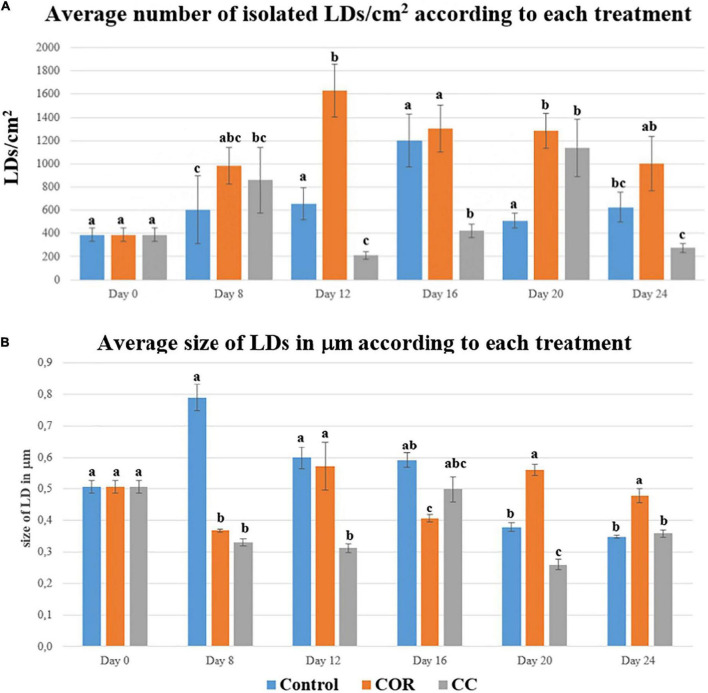
**(A)** Average number of isolated LDs/cm^2^ found in *T. baccata* cells, throughout 24 days, and maintained in Control, COR and CC conditions. Values presented are means ± SD (*n* = 10). Values followed by different letters are significantly different (*P* ≤ 0.05) according to Tukey’s honestly significant difference test. **(B)** Average size of LDs, measured in mm, found in *T. baccata* cells, throughout 24 days, and maintained in Control, COR and CC conditions. Values presented are means ± SD (*n* = 10). Values followed by different letters are significantly different (*P* ≤ 0.05) according to Tukey’s honestly significant difference test.

Figure 1A shows that the number of isolated LDs form non-treated *T. baccata* cells increased progressively and peaked (with 1200 LDs per cm^2^) on day 16, then decreased after that. Interestingly, the number of LDs fractioned from COR-treated *T. baccata* cells was significantly higher compared to their respective control and the highest number of LDs was detected for those isolated from COR-treated *T. baccata* cells on day 12, where their number per cm^2^ was 2-fold higher than the respective control. In contrast, the number of LDs isolated from CC-treated cells unsteadily varied as a function of time point. These data indicate that the treatment of *T. baccata* cells can effectively stimulate the accumulation of cellular LDs.

The largest LDs were observed in control cells at day 8 of culture (almost 0.8 mm) (Figure 1B), the average size decreasing thereafter until day 24 (0.35 mm). The LDs in COR-treated cultures were smaller than in control cells in the two first weeks after elicitation, where the average size was 1.5- and 1.4-fold higher, respectively. The smallest LDs were found in the CC cultures, except at day 16, when the size of LDs fractioned from CC-treated cells was 1.25-fold higher than those fractioned from COR cells.

In the control cultures, the number of LDs quantified within the cells (LDs/cell/cm^2^) followed a similar pattern to the isolated LDs/cm^2^, increasing steadily until day 16 (35 LDs/cell), followed by a sharp decrease at day 20 and a final increase at the end of the experiment (Supplementary Figure 3). In COR-treated cells, the number of LDs also increased until day 16, when 40 LDs/cell were observed, with few subsequent changes until day 24, when the count decreased by 50%. On the other hand, the number of cellular LDs in CC cultures was lower and seemed constant throughout the experiment, except for a slight increase at day 20 (approximately 20 LDs/cell).

**FIGURE 2:**
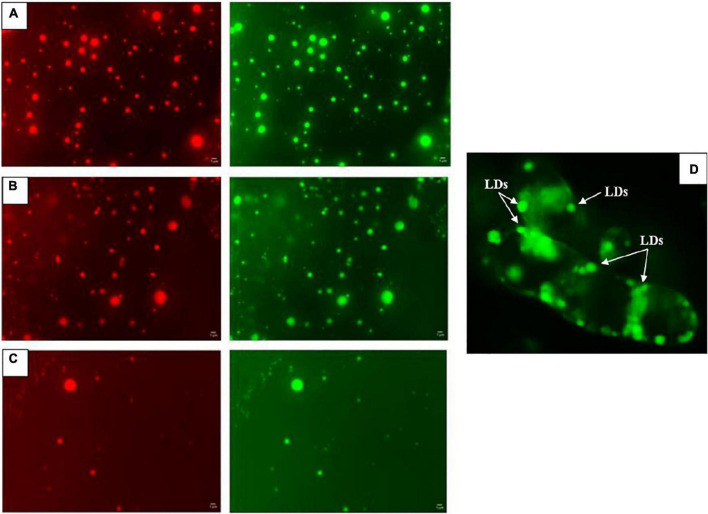
Example of detected isolated LDs under green and red fluorescence filters at 20 days of culture in COR **(A)**, CC **(B)** and Control **(C)** conditions with a fluorescence microscope at 10X ocular and 20X objective. Image **(D)** shows a clump of *T. baccata* cells with stained LDs bordering the cell wall using a green fluorescence filter at the same magnifications and 20 days of culture in COR-treated samples.

Furthermore, size and number of LDs were also examined under a fluorescence microscope as described by Hanano et al. (2022). As shown in Figure 2, the isolated LDs were detected under red and green fluorescence filters a higher magnification (200X), the images revealing a typical round shape, at 20 days of culture in COR **(A)**, CC **(B)** and control **(C)** conditions. In addition, the stained LDs are depicted close to the walls of a clump of COR-elicited *T. baccata* cells, also at day 20 and using the same magnification **(D)**.

Supplementary Figure 4 shows in detail the abundance and size of the LDs isolated from cells cultured in control, COR, or CC conditions throughout the experiment.

### Total taxane production

**FIGURE 3:**
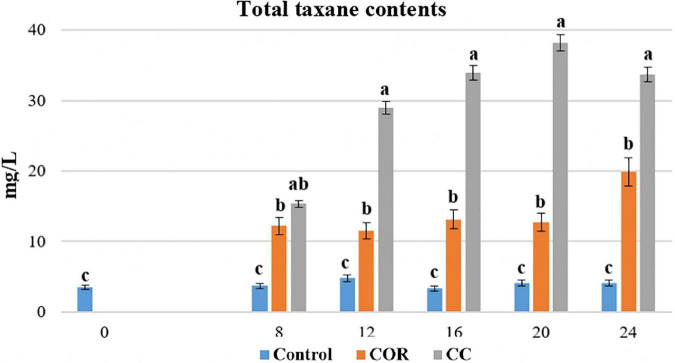
Total taxanes produced in *T. baccata* cell cultures throughout 24 days of culture in control conditions, with the addition of coronatine (COR) or supplemented with COR + β-methyl-cyclodextrins (CC). Values presented are means ± SD (*n* = 3). Values followed by different letters are significantly different (*P* ≤ 0.05) according to Tukey’s honestly significant difference test.

As has been repeatedly shown, the total taxane production, measured as the sum of 10-deacetyl baccatin III (DABIII), baccatin III (BACIII), 10-deacetyl taxol (DAT), cephalomaninne (CEPH) and paclitaxel (PTX), in unelicited *Taxus spp.* cell cultures is low (Ketchum et al., 1999; Cusido et al., 2002; Kim et al., 2005), even when they are maintained in the optimum production medium (Onrubia et al., 2013; Escrich et al., 2021). In the control conditions presented in Figure 3, the total taxane production was never higher than 5 mg/L, but the elicitor COR (1 mM) induced a clear increase on day 8 after treatment, considering both intracellular compounds and those excreted to the medium. Total taxane yield peaked on day 24 and reached 20 mg/L, corresponding to an increase of 4.8-fold compared to the control.

In the CC cultures, the total taxane production (measured as the sum of DABIII, BACIII, DAT, CEPH and PTX) increased significantly and peaked on day 20 (38.2 mg/L). At this time point, the yield in CC cultures was 3.0- and 9.3-fold higher, than in COR and control cultures, respectively

#### Partitioning of taxanes in different compartments

The contents of total and individual taxanes were determined not only in the culture medium and producer cells, but also inside the cellular LDs. The partitioning of the total taxanes between the medium, cells (excluding the LDs, which were separated from the total cell contents) and the LD fraction was determined.

As shown in Supplementary Figure 5, most of the taxanes detected at the start of the experiment were inside the cells, and, during the 24 days studied, in the control and COR-elicited cultures a high proportion remained within the cells. As expected, in the CC-treated cell cultures, a very high quantity of taxanes throughout the experiment was found secreted into the medium. The average intracellular taxanes amount in CC cultures was 3.4- and 3-fold lower and the amount of extracellular taxanes was 3.5 and 2.5-fold higher than in control and COR cultures, respectively.

**FIGURE 4:**
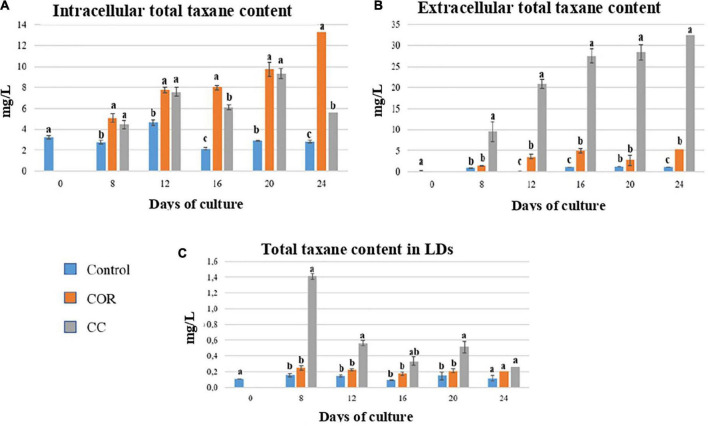
Total taxane contents found throughout the experiment inside the cells **(A)**, liquid medium **(B)** and lipidic droplets (LDs) **(C)** in *T. baccata* cell cultures in control conditions and with the addition of 1 mM coronatine (COR) or 1 mM COR plus 50 mM β-cyclodextrins (CC). Values presented are means ± SD (*n* = 3). Values followed by different letters are significantly different (*P* ≤ 0.05) according to Tukey’s honestly significant difference test.

The low content of total cellular taxanes in the control culture (Figure 4A), which did not exceed 4.5 mg/L on day 12, reflected a very low overall production. While this content increased significantly when the medium was supplemented with COR, reaching a peak on day 24 (13.3 mg/L). Although the intracellular taxanes content in the CC cell cultures was significantly higher than in the control, it still was lower than in COR cultures.

The release of total taxanes into the medium also depended on the culture conditions (Figure 4B). In control cultures, the extracellular content was low throughout the experiment, with no significant differences between samples. In COR cultures, excreted taxanes increased throughout the experiment, reaching 6.4 mg/L on day 24. The highest levels of secreted taxanes were found in the CC cultures on days 16 to 24 (approx. 28 mg/L), and they were 4.3- and 1.2-fold higher than in the COR and control cultures, respectively.

In the LDs (Figure 4C), the highest total taxane content was found on day 8 in CC cultures (1.4 mg/L), decreasing thereafter until the end of the experiment. Taxanes accumulation in LDs was low in both COR-treated and control cells, being slightly higher in the former, and did not change significantly during the culture.

#### Partitioning of total taxanes throughout the experiment

**FIGURE 5:**
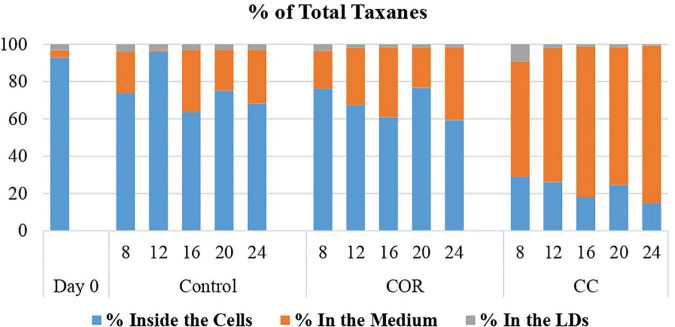
Percentage of the total taxanes found inside the cell, liquid medium and lipid droplets (LDs). *T. baccata* cell cultures were maintained for 24 days in control conditions, or with the addition of coronatine (Cor) or Cor + β-cyclodextrins (CC). Values presented are means ± SD (*n* = 3). Values followed by different letters are significantly different (*P* ≤ 0.05) according to Tukey’s honestly significant difference test.

At the beginning of the experiment, more than 90% of the total taxanes content of the cultures accumulated inside the cells (Figure 5), the percentage remaining highest in the control, followed by COR and CC cultures (68, 59, and 15%, respectively, at the end of the experiment). Correspondingly, on day 24, the percentage of taxanes in the culture medium was lower in the control (29%) compared to COR (39%) and CC (85%) cultures.

In the LDs, the highest percentage of total taxanes was found on day 8 in all the conditions studied (4.2, 3.4, and 9.2% in control, COR and CC cultures, respectively).

#### Individual taxane production

Among the individual taxanes (DABIII, BACIII, DAT, CEPH, and PTX), very clear differences were observed in their partitioning between the three culture fractions (cells after separating the LDs, the medium, and LDs), mainly due to their chemical structures, as well as the treatment and development stage of the cell culture.

**FIGURE 6:**
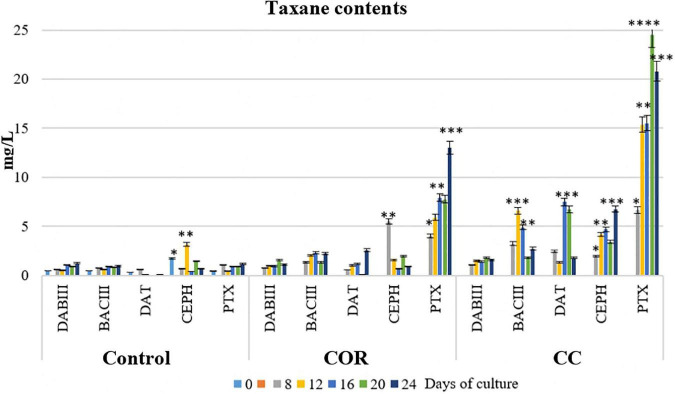
Contents of the different taxanes studied (accumulated in the cells, in the liquid medium and in LDs) throughout the experiment in *T. baccata* cell cultures in elicited and control cultures. COR: 1 mM coronatine; CC: 1 mM coronatine + 50 mM β-methyl-cyclodextrins. Values presented are means ± SD (*n* = 3). Values followed by different symbols are significantly different (*P* ≤ 0.05) according to Tukey’s honestly significant difference test.

As expected, the contents of the different taxanes were extremely low at the start of the experiment prior to elicitation (time 0) (Figure 6), being no higher than 0.5 mg/L, except for CEPH (1.7 mg/L). Here, in control conditions, the content of taxanes without a side chain remained low throughout the experiment, never exceeding 1.2 mg/L. The content of taxanes bearing a side chain was also low but variable: DAT was almost non-existent, PTX reached the highest level at the end of the culture (1.2 mg/L), whereas CEPH peaked on day 12 (3.2 mg/L).

In the COR-elicited cultures (Figure 6), taxane production was significantly enhanced. DABIII, BACIII and DAT generally increased throughout the experiment, although the highest levels remained low (1.1 mg/L, 2.1 mg/L, and 2.6 mg/L, respectively, on day 24). The highest content of CEPH was detected on day 12 (5.5 mg/L), decreasing thereafter, whereas the content of PTX increased constantly throughout the experiment, reaching 13 mg/L on day 24. Compared to the control, BACIII, DAT, CEPH, and PTX levels on day 24 in COR-elicited cultures were 2.4-, 37.0-, 1.5-, and 11.0-fold higher, respectively. The addition of COR did not significantly change DABIII production, probably because it was quickly converted to BACIII or other related taxanes. The contents of DAT clearly increased in comparison to the control, although the levels were similar to those of BACIII at the end of the culture and DABIII in the first time points. Taken together, PTX was the taxane with the strongest response to the action of COR.

The co-treatment with COR and β - CDs (Figure 6) induced a clear increase in the production of taxanes, more particularly PTX. At the end of the culture, the PTX content was 21 mg/L, i.e., almost 1.7-fold higher than in COR-elicited cultures and almost 18-fold higher than in the control.

#### Partitioning of individual taxanes in different compartments

The partitioning of taxanes between different compartments of a culture system, in this case cells, culture medium, and LDs, is of interest, not only from the point of view of basic research, but also for the development of strategies to enhance taxane excretion from the producer cells. The accumulation of taxanes in the medium facilitates their extraction and avoids their toxic effects on the active cell metabolism.

**FIGURE 7:**
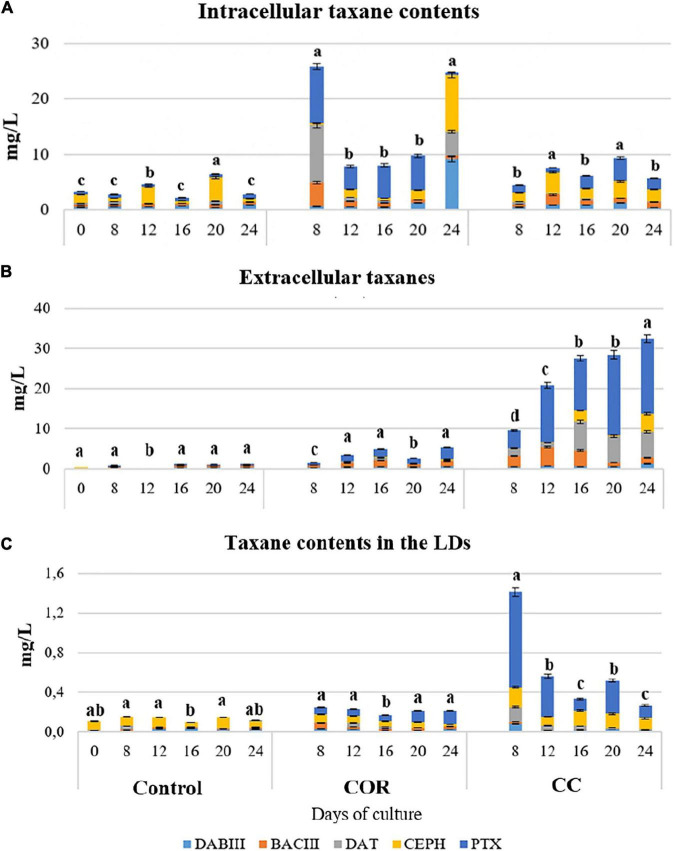
Taxane content (expressed as mg L^–1^) found inside the cells **(A)**, in the culture medium **(B)** and within the LDs **(C)** of *T. baccata* cell cultures when maintained for 24 days in the optimum medium for production complemented or not with elicitors. COR: 1 mM coronatine; CC: 1 mM coronatine + 50 mM β-CDs; DABIII: 10-deacetylbaccatin III; BACIII: baccatin III; DAT: 10deacetylpaclitaxel; CEPH: cephalomannine; PTX: paclitaxel. Values presented are means ± SD (*n* = 3). Values followed by different letters are significantly different (*P* ≤ 0.05) according to Tukey’s honestly significant difference test.

As shown in Figure 7A, the main intracellular taxane on day 0 was CEPH (almost 50% of the total taxanes), confirming that the studied cell line was naturally a high CEPH producer (Vidal-Limon et al., 2018). In control cultures, CEPH represented 66% of the total taxanes accumulated inside the cells on day 12, whereas the other taxanes ranged from 10 to 20% at different time points, except for DAT, which constituted only 1-2% on days 12 and 16. The maximum intracellular accumulation of PTX (4.2 mg/L) was on day 20, representing 44% of the total taxanes.

Similar percentages of intracellular taxanes accumulation were found in cultures treated with COR or CC (Figure 7A). The major taxanes were also CEPH and PTX, especially in the second half of the experiment, the levels reaching 6.7 mg/L and 20 mg/L, respectively, in CC cultures at day 20, which represented 33 and 45% of the total taxanes production.

As shown in Figure 7B, in control conditions, none of the taxanes in the culture medium were detected at levels above 0.5 mg/L, and in COR-treated cultures, a slight increase was observed only in BACIII and PTX, which reached 1.6 mg/L at day 16 and 2.9 mg/L on day 24, respectively. A significant increase in taxane release observed in CC cultures was particularly striking for DAT, CEPH and PTX from day 20 to 24, whereas the maximum excretion of BACIII took place on day 12. The excretion capacity of cells varies during culture, depending on the development and metabolic stage of cells, taxane production levels, and individual taxanes (Seki et al., 1997; Kim et al., 2005). It is not surprising, therefore, to find fluctuations in taxanes excretion. Nevertheless, as shown in Figure 7B, the major taxane found in the medium of CC cultures was PTX, with levels of 20 mg/L on day 20, which corresponded to 70% of the total taxanes in the culture medium. The experimental conditions associated with the highest PTX production were also those that induced its maximum excretion. It was therefore once again patent that the capacity of the elicitor COR to activate PTX biosynthesis and of β-CDs to form inclusion complexes with lipophilic compounds such as PTX and related taxanes can be jointly harnessed to achieve high PTX-producing cell culture systems.

It is striking that the main taxanes found in the LDs (Figure 7C), PTX followed by CEPH, both have side chains. In control conditions, taxanes contents were low, the highest being found on day 20, when CEPH (0.111 mg/L) represented 75% of the total taxanes, whereas PTX was not detected at any time point. In COR-treated cultures, CEPH (0.05 mg/L) and PTX (0.11 mg/L) corresponded to 26% and 53% of the total taxanes accumulated in LDs on day 20. In CC cultures, taxanes levels in LDs were highest at day 8, when CEPH (0.14 mg/L) and PTX (0.33 mg/L) represented 27 and 64%, respectively, of the total taxanes; the content decreased thereafter, although remaining higher than in control and COR cultures.

**FIGURE 8:**
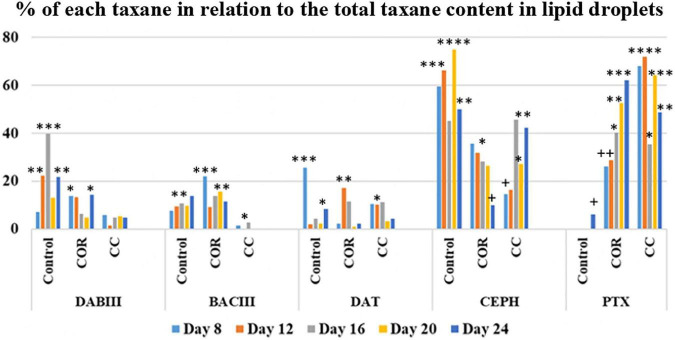
Percentage of each taxane in relation to the total accumulated in the LDs, in *T. baccata* cell cultures when maintained for 24 days in the optimum medium for production complemented or not with elicitors. COR: 1 mM coronatine; CC: 1 mM coronatine + 50 mM β-CDs; DABIII: 10-deacetylbaccatin III; BACIII: baccatin III; DAT: 10deacetylpaclitaxel; CEPH: cephalomannine; PTX: paclitaxel. Values presented are means ± SD (*n* = 3). Values followed by different symbols are significantly different (*P* ≤ 0.05) according to Tukey’s honestly significant difference test.

Comparing the percentage of each taxane in relation to the total taxanes accumulated inside the LDs (Figure 8) reveals that DABIII was mainly sequestered in control cells (40% on day 20), followed by COR-treated cells, although at much lower levels. BACIII was detected in LDs of control and COR-treated cells (22% maximum) and was practically absent in those of CC cultures. LDs in all the studied conditions contained DAT, especially in the control cultures at the beginning of the experiment (25%) and in COR cultures from days 12 to 16 (12 to 17%). A high proportion of CEPH was detected in LDs regardless of treatment: in the control throughout the study (50 - 75%); in COR cultures (26 - 35%) except for a decrease at the end; and in CC conditions in the second half of the culture period (approximately 40%). The main taxane accumulated in LDs in COR and CC cultures was PTX (50 - 70%), the proportion declining slightly toward the end (50 - 60%).

### Transcripts profile of paclitaxel biosynthetic genes

The expression levels of genes encoding enzymes involved in PTX biosynthesis were determined by RT-qPCR and their relationship with the pattern of taxane production was analyzed. The target genes were *TXS*, encoding taxadiene synthase, *T7βOH*, encoding taxadiene 17-β-hydroxylase, *BAPT*, encoding baccatin III-3-amino-13-phenylpropanoyltransferase, and *DBTNBT*, encoding 3′N-benzoyltransferase. The expression level of the *ABC* transporter gene was also studied, as ABC transporters play an important role in the removal of secondary metabolites from the active cytoplasm to avoid their toxic effects on cell viability (Fornale et al., 2002; Sun et al., 2013).

The genes expression was determined from 1 h to 3 days after elicitation. For each gene, the expression levels are expressed in relation to those found in cell suspensions at day 12 of culture in the optimal GM (reference value = 1), as described in Material and methods.

**FIGURE 9:**
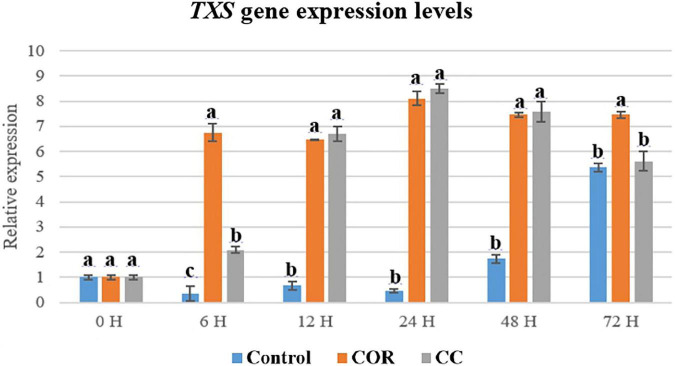
Relative expression of TXS gene in *T. baccata* cell cultures grown in Control conditions and elicited with 1 mM coronatine (COR) or 1 mM coronatine + 50 mM methyl-β-cyclodextrins (CC). The relative gene expression levels were normalized with respect to the same cell line growing for 12 days in the growth medium (GM) without elicitors (reference value = 1). Values presented are means ± SD (*n* = 3). Values followed by different letters are significantly different (*P* ≤ 0.05) according to Tukey’s honestly significant difference test.

The transcripts of *TXS* gene (Figure 9) in control cells increased steadily until 48 h of culture, achieving the highest level (5 fold at 72 h. In COR-treated cultures, it was very high from 6 h until the end of the study, peaking at 24 h (8 fold), whereas in CC cultures, accumulation was clearly enhanced from 12 h, also achieving the highest level at 24 h (8 fold). Differences in genes transcripts in both treatments was not significantly different except at 6 h and 72 h, when COR had a greater effect.

The active transcription of *TXS* gene is crucial for the biosynthesis of all the intermediates of the PTX biosynthetic pathway, as it encodes the enzyme that catalyzes the cyclization of GGPP to form taxa-4(5),11(12)-diene, the first taxane with the taxane skeleton.

**FIGURE 10:**
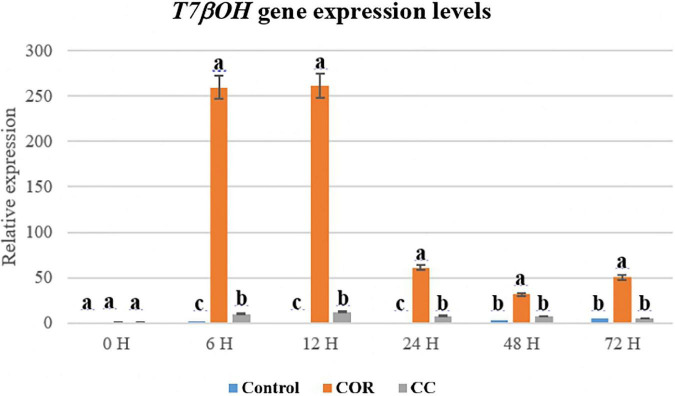
Relative expression of the T7βOH gene in *T. baccata* cell cultures grown in Control conditions and elicited with 1 mM coronatine (COR) or 1 mM coronatine + 50 mM methyl-β-cyclodextrins (CC). Values presented are means ± SD (*n* = 3). Values followed by different letters are significantly different (*P* ≤ 0.05) according to Tukey’s honestly significant difference test.

Transcripts of *T7βOH* gene was very high in COR-treated cultures from 6 h to 12 h (260 fold), decreasing thereafter (Figure 10). At its highest, the expression of this gene in control and CC cultures was 260- and 250-fold lower than in cells elicited only with COR. Supplementing the cultures with CC also increased the expression level, which at its highest at 12 h was 12-fold higher than in control cells. In general, *T7βOH* gene transcription was much higher compared to the *TXS* gene.

**FIGURE 11:**
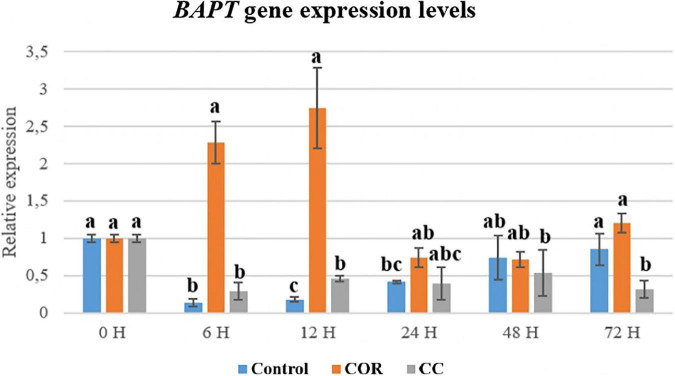
Relative expression of the BAPT gene in *T. baccata* cell cultures grown in Control conditions and elicited with 1 mM coronatine (COR) or 1 mM coronatine + 50 mM methyl-β-cyclodextrins (CC). Values presented are means ± SD (*n* = 3). Values followed by different letters are significantly different (P ≤ 0.05) according to Tukey’s honestly significant difference test.

The *BAPT* gene is involved in the formation of side chain-bearing taxanes such as PTX, CEPH and probably DAT, although the biosynthetic steps leading to the latter are still not fully elucidated. Expression of the *BAPT* gene (Figure 11) was most clearly enhanced by COR, mainly from 6 h to 12 h of elicitation (2.3- and 2.8-fold higher compared to control and CC cultures, respectively), although to a much lower extent than the *T7βOH* gene.

**FIGURE 12:**
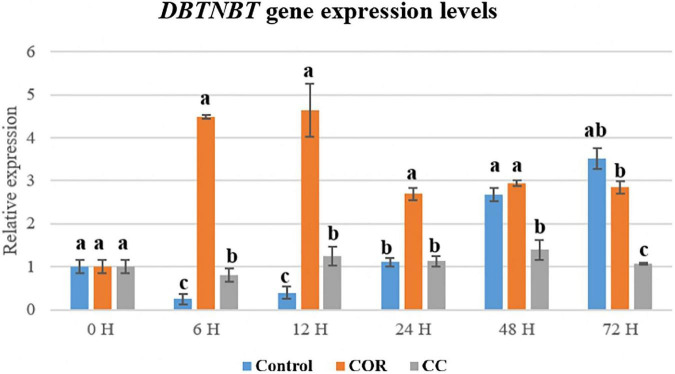
Relative expression of the DBTNBT gene in *T. baccata* cell cultures grown in Control conditions and elicited with 1 mM coronatine (COR) or 1 mM coronatine + 50 mM methyl-β-cyclodextrins (CC). Values presented are means ± SD (*n* = 3). Values followed by different letters are significantly different (*P* ≤ 0.05) according to Tukey’s honestly significant difference test.

The expression pattern observed for the *DBTNBT* gene was similar to that of *BAPT* (Figure 12). The product of this gene, 3’N-benzoyltransferase, is specifically involved in the formation of PTX, since CEPH and DAT have a tigloyl group at the C-3’ position of the side chain, or do not have any group at this position, respectively. Thus, the *DBTNBT* is the only gene exclusive to PTX biosynthesis. Expression of the *DBTNBT* gene was highest in the COR-treated cultures from 6 h to 12 h, decreasing thereafter until 72 h, being 4.5- and 4.7-fold higher than in control and CC conditions, respectively, and always greater than in the CC-treated cells.

It is noteworthy that the *ABC* gene (Supplementary Figure 6) was more expressed in COR-treated than in control cultures at 6 h after elicitation, as overall its transcripts levels were highest in control cells and lowest in CC cultures. *In vitro*-cultured *Taxus spp.* cells probably have an inherent amount of ABC transporters that allow potentially toxic metabolites such as taxanes to be moved to a separate compartment or outside the cell.

## Discussion

Biomass formation and taxane production was studied in *T. baccata* cell cultures treated with the elicitor coronatine (COR) or COR plus methyl-β-cyclodextrins (CC), and compared with untreated control cultures. Additionally, the expression pattern of genes involved in paclitaxel (PTX) biosynthesis was analyzed. Due to the recently described role of lipid droplets (LDs) in the storage and trafficking of lipid compounds in cells, the characteristics and taxane accumulation capacity of these organelles was also studied.

*Taxus baccata* cell cultures were grown in a medium optimized for taxane production (Palazon et al., 2003), which also permitted a relatively good growth. However, biomass formation was limited by the elicitation treatments, as observed previously, including for COR (Onrubia et al., 2013; Sabater-Jara et al., 2014; Ramirez-Estrada et al., 2015; Vidal-Limon et al., 2018; Escrich et al., 2021). In studies with *T. media* and *T. globosa*, we have found that β-CDs can counteract the adverse effect of COR on growth (Ramirez-Estrada et al., 2015), but this was not observed here in the *T. baccata* cell line. Besides the negative effect of elicitors on the biomass formation in *Taxus* cell suspensions (Furmanova and Syklowska-Baranek, 2000; Onrubia et al., 2013; Sabater-Jara et al., 2014), the toxicity of PTX and other taxanes impedes cell viability and growth (Qiao et al., 2003; Kim et al., 2005; Exposito et al., 2009). In the present study, the highest intracellular content of PTX was found in COR- and CC-treated cultures after 20 days (64 and 45%, respectively, of the total cell-associated taxanes), an amount sufficient to inhibit growth. The growth response of *Taxus* spp cultures to the addition of elicitors has been extensively studied. We can highlight the relevance of the studies carried out by Sykłowska-Baranek et al. (2018, 2019), with elicited cultures of *T. media* hairy roots. The addition of elicitors such a methyl jasmonate together with sodium nitroprusside, L-phenylalanine and additional sucrose, to *T. media* hairy root cultures overexpressing the *TXS* gene, was not negative for growth (measured as fresh weight), especially when an aerated perfluorodecalins phase was added to the culture medium. Contrary, the same hairy root line, when growing in an elicited culture medium with degassed perfluorodecalins phase presented a clear reduction of growth. These studies corroborated the fact that the response of taxane biotechnological production systems to elicitation depends not only on factors related to the elicitor itself (type, concentration, duration of elicitation, etc.) but also on the species, cell/organ cultures and state of development of the culture.

Our results indicate that the fractioned LDs from the non-treated *T. baccata* cells had all the structural characteristic that typically found in other plant LDs (Hanano et al., 2006, 2016). More pertinently, our results come in line with our recently results describing the cellular LDs from *T. media* cells suspension (Hanano et al., 2022). In this regard, it is also now apparent that LDs are not only present in storage tissues such as seeds and pollen grains, but are also found in most, if not all, cell types in eukaryotes (Murphy, 2012). The roles of cellular LDs in plant cell have been recently illustrated with a particular focus on their implication in trafficking and secretion of PTX *in vitro* as well as in planta (Hanano et al., 2022). Even more, the application of plant LDs in the sequestration of hydrophobic environmental contaminates was rigorously administrated (Boucher et al., 2008; Hanano et al., 2016).

The highest total taxane production was obtained in cultures supplemented with COR and even more so, COR + β-CDs, the yields being more than 3- and 9-fold higher, respectively, than in the control. The CC cultures stood out, however, in that about 80% of the total taxanes were found in the medium throughout the experiment, whereas in control and COR cultures, about 60-70% were cell-associated. The excretion behavior of cells can vary between plant species, culture conditions, and cell lines. Thus, in a *T. media* cell line, the addition of β-CDs together with MeJA or COR to the culture medium induced an excretion of more than 80% and 90%, respectively, of the total taxanes produced (Sabater-Jara et al., 2014; Ramirez-Estrada et al., 2015). Although β - CDs do not act as an elicitor in *Taxus* cell cultures (Sabater-Jara et al., 2014; Ramirez-Estrada et al., 2015), their ability to sequester compounds favors a high production of taxanes, especially PTX. Recently, in *Coryllus avellana* cell suspensions, Farhadi et al. (2020) showed that β-CDs, either alone or in combination with fungal elicitors, induced a high secretion of PTX to the medium, whereas more than 50% remained inside the untreated *C. avellana* cells. Differences in the excretion capacity of cells is also patent between unelicited cell cultures. PTX release reached 66% in *T. cuspidata* cells according to Fett-Neto et al. (1994), and 90% in a *T. baccata* cell line studied by Hirasuna et al. (1996). We observed that unelicited *T. baccata* and *T. wallichiana* cells grown in a bioreactor had an excretion capacity of 20% and 52%, respectively, which again demonstrates the variation between species and cell lines (Navia-Osorio et al., 2002). The fluctuating capacity of cells to release PTX during the growth period was described by Seki et al. (1997) in free and immobilized cell cultures of *T. cuspidata*. The release of taxanes from the cells to the medium is desirable, as it avoids toxic effects in the cells, enhances biosynthesis (Navia-Osorio et al., 2002), and facilitates downstream processing.

Patterns of taxane accumulation in *Taxus spp*. are affected by environmental and genetic factors and can vary between cell lines. Individual taxane levels (cell-associated + extracellular + in LDs) differed between the *T. baccata* cultures according to the treatment. In the control, in ascending order, they were DAT (0.2 mg/L - 0.6 mg/L), BACIII (0.8 mg/L - 1 mg/L), DABIII (0.5 mg/L - 1.2 mg/L, PTX (0.5 mg/L - 2 mg/L) and CEPH (0.5 mg/L - 3.2 mg/L). Also in ascending order, in the COR-treated cultures they were DAT (1.2 mg/L), DABIII (1.6 mg/L), CEPH (2.1 mg/L), BACIII (4.4 mg/L) and PTX (8 mg/L), and in CC cultures, DABIII (1.8 mg/L), BACIII (6.6 mg/L), CEPH (6.8 mg/L), DAT (7.5 mg/L) and PTX (25 mg/L). These results show that COR and even more so, COR + β-CDs are highly effective treatments for increasing taxane production in cell cultures. The fact that DABIII and BACIII levels were always significantly lower than those of PTX indicates that the treated cells were metabolically active and that these precursors were readily converted to PTX. Notably, the main taxanes in the CC cultures were DAT, CEPH and PTX (all bearing the phenylisoserine side chain), suggesting that the attachment of the side chain at C13 of BACIII is probably not a flux-limiting step in PTX biosynthesis. Although doubts remain about whether DAT is formed from BACIII or if it is a transformation product of PTX, significant levels of this taxane have been reported in *T. cuspidata* (Zhang et al., 2010) and *T. wallichiana* (McLaughlin et al., 1981), in cell suspensions of *T. baccata* (Mroczek et al., 2000; Palazon et al., 2003; Onrubia et al., 2010) and *T. media* (Cusido et al., 2002; Exposito et al., 2010), and in hazel cell cultures (Gallego et al., 2015). DAT and its 7-xylosyl derivative are currently considered as suitable precursors for the semi-synthesis of PTX, because they are found in greater amounts than PTX in yew tree bark, and DAT can be easily transformed into PTX by acetylation at the C10 position. CEPH is usually found in *Taxus spp.* cell cultures (Ramirez-Estrada et al., 2015; Vidal-Limon et al., 2018), but in the present study, its accumulation was significantly lower than that of PTX (approximately 3.5-fold lower when its production was highest). Like PTX, CEPH is formed from BACIII and β-phenylalanoil-CoA. Consequently, these low levels suggest a rapid conversion to other taxanes or a low efficiency of tigloyl transferase, which catalyzes the last step in CEPH biosynthesis, the tigloylation of the side chain.

In CC cultures, the percentage of each taxane in the culture medium at the peak of production (normally 20 or 24 days after elicitation) was generally very high: DABIII (30.3%), BACIII (61.8%), DAT (91%), CEPH (55.7%) and PTX (81.6%) (measured as the sum of content in the cells, liquid medium and LDs). Except for DABIII, more than half of each taxane yield was released from the cells to the medium, especially the side chain-bearing DAT and PTX.

Responsible for this high excretion of taxanes from the producer cells is β-CD (methyl-β-cyclodextrin). Its chemical structure allows taxanes to be trapped within the β-CDs, forming inclusion complexes. Very little is known about the capacity of CDs to penetrate the wall of plant cells. In differentiated plant tissue, the cell wall pore diameter (approximately 10⋅10^–9^ cm) is too small for β-CDs (1.53⋅10^–7^ cm) to reach the cell membrane. However, in actively growing cell suspensions, the primary cell walls are very thin, cemented together by the middle lamella, and although highly cohesive, they have a strong capacity to undergo rapid modification during cell division and elongation (Chambat et al., 1984). Regarding animal cells, rhodamine-labeled random methyl-β-CDs are reported to enter human intestinal epithelial Caco-2 cells and HeLa cells by mechanisms of endocytosis (Réti-Nagy et al., 2015; Haimhoffer et al., 2019). Réti-Nagy et al. (2015) also showed that PTX penetrated animal cells together with β-CDs, indicating that these inclusion complexes can pass through the plasma membrane. To the best of our knowledge, there is no available information about the mechanisms of entry and exit of β-CDs in plant cells, which may differ according to the cell type or several may operate to varying degrees. However, it is feasible that plant cells use similar mechanisms to animal cells. As previously shown (Sabater-Jara et al., 2014), the capacity of β-CDs to form inclusion complexes with taxanes both inside and outside cells, avoiding their enzymatic degradation, toxic effects and retro-inhibition processes, is probably responsible for the high production and excretion capacity of *Taxus* cell cultures co-supplemented with COR and β-CDs.

Notably, the main taxanes found in LDs were CEPH and PTX, which only differ in one substituent on the side chain, being either a tigloyl group or a benzyl ring, respectively. The other taxanes studied were also found inside the LDs, but to a much lower extent. Hanano et al. (2022) have reported that LDs can sequester and traffic hydrophobic molecules in animal and plant cells (Hanano et al., 2016, 2018). They recently detected PTX in the LD fraction in *T. media* cell cultures, and found that the number of LDs increased with PTX production. This increase was synchronized with the appearance of a 27 kDa-caleosin, suggesting that this protein, which is an important constituent and stabilizing element of LDs, may play a role in the biosynthesis and trafficking of PTX. Hanano et al. (2022) also studied the ability of LDs from *T. media*, date palm seeds and *Arabidopsis* to sequester PTX *in vitro.* They found that about 96% of the PTX in solution accumulated in the LDs of all the species studied. Interestingly, the PTX uptake depended on the pH of the solution, and declined in acidic conditions. Our results are consistent with those of Hanano et al. (2022), as the percentage of PTX and CEPH inside the LDs in our experiment was much lower than 96% and the pH of the culture medium, although variable, was always acidic (pH 5.8).

Some of the PTX and other taxanes found in the liquid medium would have been released from the cells when LDs were fused with the cell membrane. However, further studies are needed to determine to what extent the excreted taxanes originate from sequestration by CDs or LDs, or the activity of ABC transporters in the cell membrane.

The expression of different genes involved in PTX biosynthesis is affected variably by the culture treatments and depends on the time the elicitor and β-CDs are in contact with the cells (Onrubia et al., 2013; Sabater-Jara et al., 2014; Sanchez-Muñoz et al., 2018). The high transcript level of the TXS gene in COR- and CC-treated cultures suggests that the precursor taxa-4 (5), 11 (12)-diene was formed in sufficient quantity to increase the production of downstream taxanes whose formation depends on the action of taxadiene synthase. The same was observed for the T7-β-OH gene, which is also required for the biosynthesis of all the target taxanes. Moreover, the T7-βOH gene was generally more expressed than the TXS gene. All the hydroxylases identified in *Taxus* spp., including T7-β-OH, are cytochrome P450-dependent enzymes with comparable mechanisms of action (Kaspera and Croteau, 2006). This can explain their shared strong response to elicitation, which markedly upregulates their encoding genes, as we have observed in previous studies with *T. baccata* and *T. media* cell cultures treated with various elicitors (Onrubia et al., 2013; Sabater-Jara et al., 2014; Ramirez-Estrada et al., 2015; Sanchez-Muñoz et al., 2018; Vidal-Limon et al., 2018; Escrich et al., 2021).

BAPT and DBTNBT gene expression was enhanced by COR but not by the CC treatment. The very high PTX production in the CC cultures (24.5 mg/L; almost 25-and 2-fold higher than in control and COR cultures, respectively) was therefore not only a direct consequence of BAPT and DBTNBT up-regulation. The accumulation of CEPH and DAT, taxanes whose biosynthesis involves the BAPT gene, never surpassed 7 mg/L in any of the elicited cultures. In the case of CEPH, this suggests a rapid conversion or low efficiency of tigloyl transferase, which as mentioned catalyzes the last step of its formation. However, to fully understand these results, the biosynthesis of these two taxanes requires further elucidation. Other factors to take into account are the possible epigenetic changes undergone by genes when cells are cultured in *in vitro* conditions, especially long-term. Sanchez-Muñoz et al. (2018) showed that increased methylation of the Y-patch promoter region of the BAPT gene reduced the elicitation response over time, leading to a lower expression level of this gene and taxane production. The same was found to have occurred to the DBTNBT gene (data not shown), whereas other genes such as TXS and various hydroxylase-encoding genes showed few epigenetic changes. From all these results, it may be inferred that the BAPT and DBTNBT genes control flux-limiting steps of the PTX biosynthetic pathway in *T. baccata* cell cultures, although the levels of these taxanes were clearly increased by the presence of COR and β-CDs. The clear inhibition of the ABC gene in CC cultures could be due to the presence of β-CDs, which are reported to inhibit cell efflux pumps in intestinal Caco-2 cells (Réti-Nagy et al., 2015). The phenomenon of ABC transporter inhibition by CDs and their derivatives has been widely investigated in animal cancer cells (Coisne et al., 2016; Yin et al., 2018), but to our knowledge it has not been explored in plant cell cultures.

This study confirms that elicitation with COR is an efficient strategy to increase PTX production in *T. baccata* cell cultures, which can be further enhanced by co-supplementation with β-CDs to promote the release of PTX from the producer cells. The importance of LDs in the accumulation and trafficking of PTX was demonstrated, as these organelles increased in number after the elicitation of cell cultures, and the anticancer agent was the main taxane stored inside.

## Data availability statement

The raw data supporting the conclusions of this article will be made available by the authors, without undue reservation.

## Author contributions

EP-M performed the experiments and contributed to the design of the experimental work. JP and RC conceived and designed the experimental work and wrote the manuscript. MB and EM supervised the work, read and commented the manuscript. AH wrote, read, commented, and edited the manuscript. All authors contributed to the article and approved the submitted version.

## Footnotes

1
https://www.precedenceresearch.com/paclitaxel-injection-market

## Supplementary material

Supplementary Figure 1 Time courses of biomass production (measured as fresh weight) of *T. baccata* cell suspensions cultured for 24 days in the production medium in control conditions or with the addition of 1 mM COR (coronatine) or 1 mM COR + 50 mM β-CDs (methyl-β-cyclodextrins) (CC). In all cases, the inoculum consisted of 300 g/L of cells. Data represent average values from three separate experiments ± SD.

Supplementary Figure 2 Time courses of biomass production (measured as dry weight) of *T. baccata* cell suspensions cultured for 24 days in the production medium in control conditions or with the addition of 1 mM coronatine (COR) or 1 mM COR + 50 mM β-CDs (methyl-β-cyclodextrins) (CC). Data represent average values from three separate experiments ± SD.

Supplementary Figure 3 Average number of LDs/cell/cm^2^ found in *T. baccata* cells, throughout 24 days, and maintained in Control, COR and CC conditions. Values presented are means ± SD (*n* = 20). Values followed by different letters are significantly different (*P* ≤ 0.05) according to Tukey’s honestly significant difference test.

Supplementary Figure 4 Example of the size and abundancy of the isolated LDs from *T. baccata* cells cultured in Control, COR and CC conditions, throughout of the experiment by using a fluorescence microscope at 10X ocular and 10X objective. Size and abundance were calculated using ImageJ software and expressed as means ± SD (*n* = 10).

Supplementary Figure 5 Average sum of the different target taxanes found inside the cells, culture medium, and lipidic droplets (LDs) throughout the 24 days of the study. *T. baccata* cell cultures were maintained in control conditions or with the addition of 1 mM coronatine (COR) or 1 mM coronatine + 50 mM β-cyclodextrins (CC). Values presented are means ± SD (*n* = 3). Values followed by different letters are significantly different (*P* ≤ 0.05) according to Tukey’s honestly significant difference test.

Supplementary Figure 6 Relative expression of the ABC gene in *T. baccata* cell cultures grown, for 72 h, in Control conditions and elicited with 1 mM coronatine (COR) or 1 mM coronatine + 50 mM methyl-β-cyclodextrins (CC). Values presented are means ± SD (*n* = 3). Values followed by different letters are significantly different (*P* ≤ 0.05) according to Tukey’s honestly significant difference test.

Supplementary Table 1 Sequences of the primers used to amplify the genes by RT-qPCR.
