# Supplementary figures and images for: Insights into the control of taxane metabolism: Molecular, cellular, and metabolic changes induced by elicitation in Taxus baccata cell suspensions
Source: Front Plant Sci. 2022 Jul 29;13:942433. doi: 10.3389/fpls.2022.942433 (PMC9372332; doi:10.3389/fpls.2022.942433)

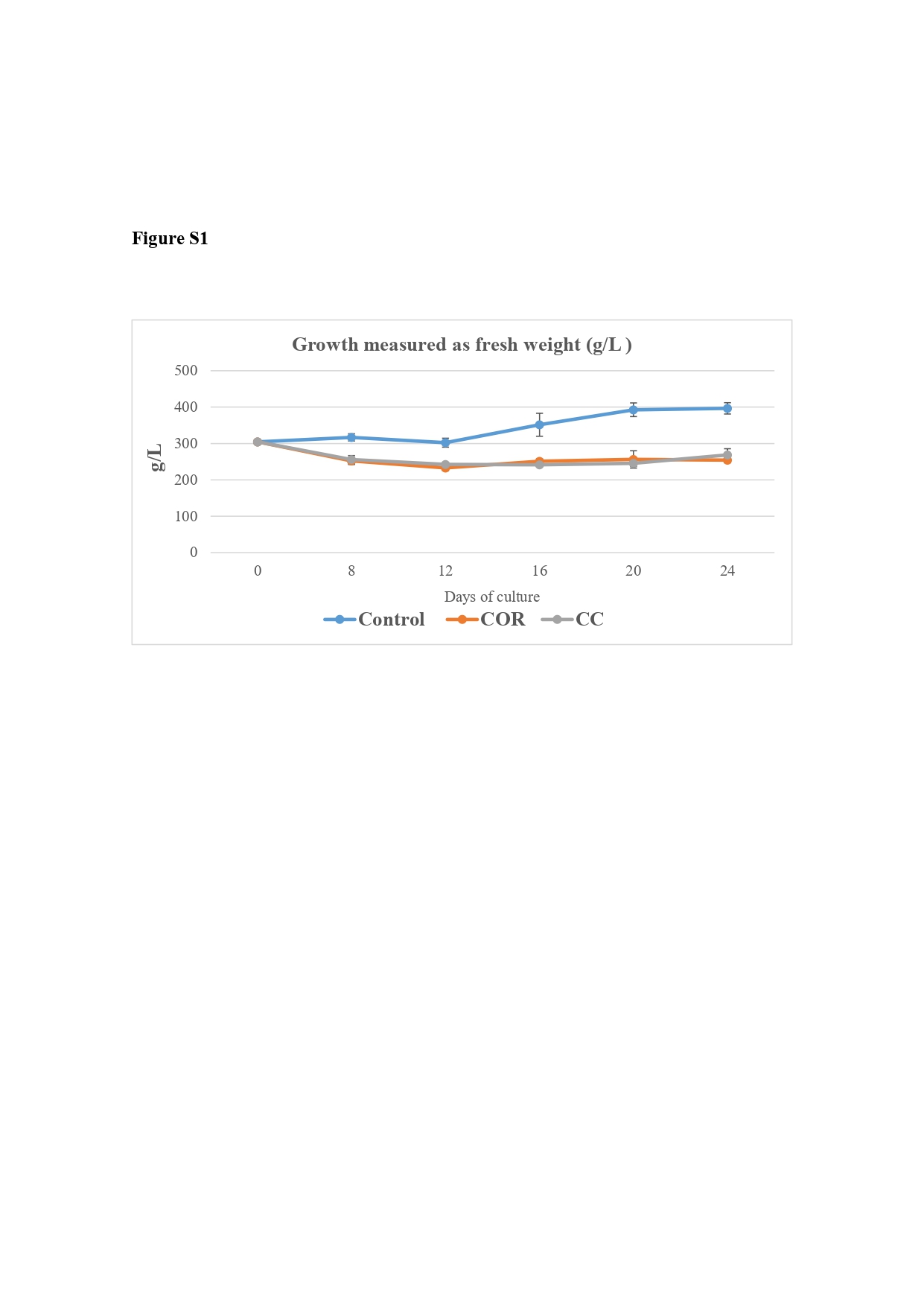

Supplement: Supplementary Figure 1 — Time courses of biomass production (measured as fresh weight) of T. baccata cell suspensions cultured for 24 days in the production medium in control conditions or with the addition of 1 mM COR (coronatine) or 1 mM COR + 50 mM β-CDs (methyl-β-cyclodextrins) (CC). In all cases, the inoculum consisted of 300 g/L of cells. Data represent average values from three separate experiments ± SD. [file Image_1.jpg]

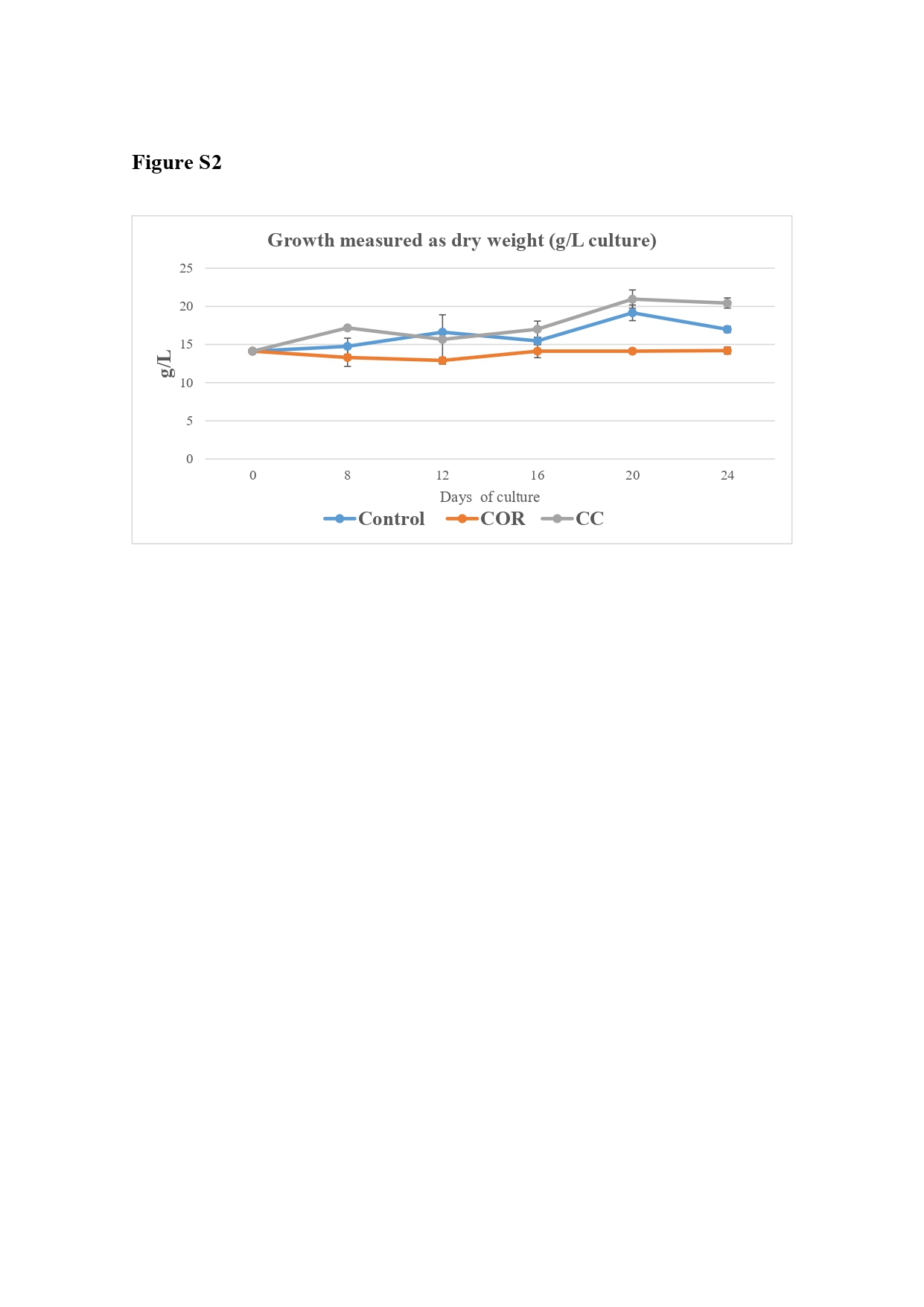

Supplement: Supplementary Figure 2 — Time courses of biomass production (measured as dry weight) of T. baccata cell suspensions cultured for 24 days in the production medium in control conditions or with the addition of 1 mM coronatine (COR) or 1 mM COR + 50 mM β-CDs (methyl-β-cyclodextrins) (CC). Data represent average values from three separate experiments ± SD. [file Image_2.jpg]

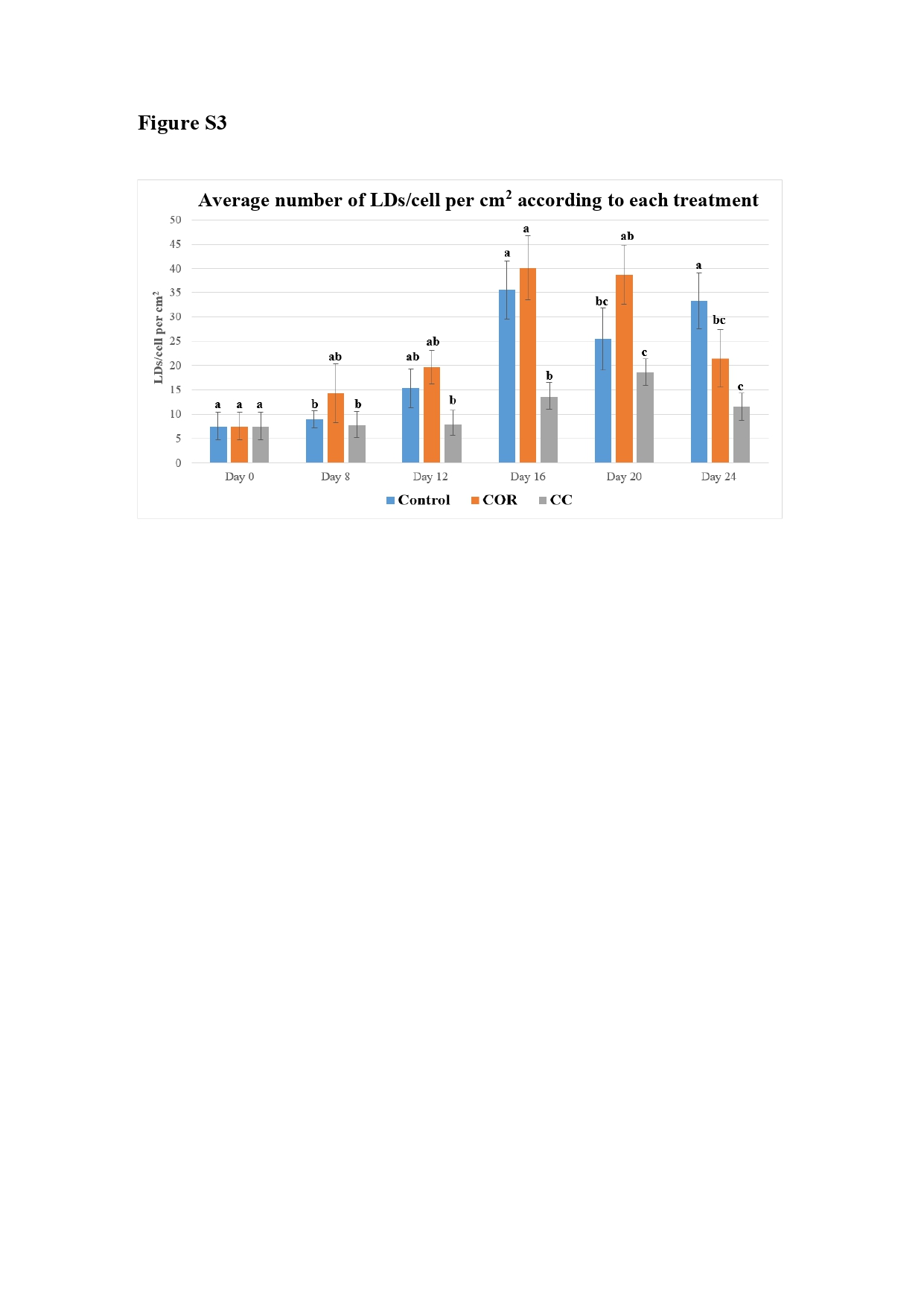

Supplement: Supplementary Figure 3 — Average number of LDs/cell/cm2 found in T. baccata cells, throughout 24 days, and maintained in Control, COR and CC conditions. Values presented are means ± SD (n = 20). Values followed by different letters are significantly different (P ≤ 0.05) according to Tukey’s honestly significant difference test. [file Image_3.jpg]

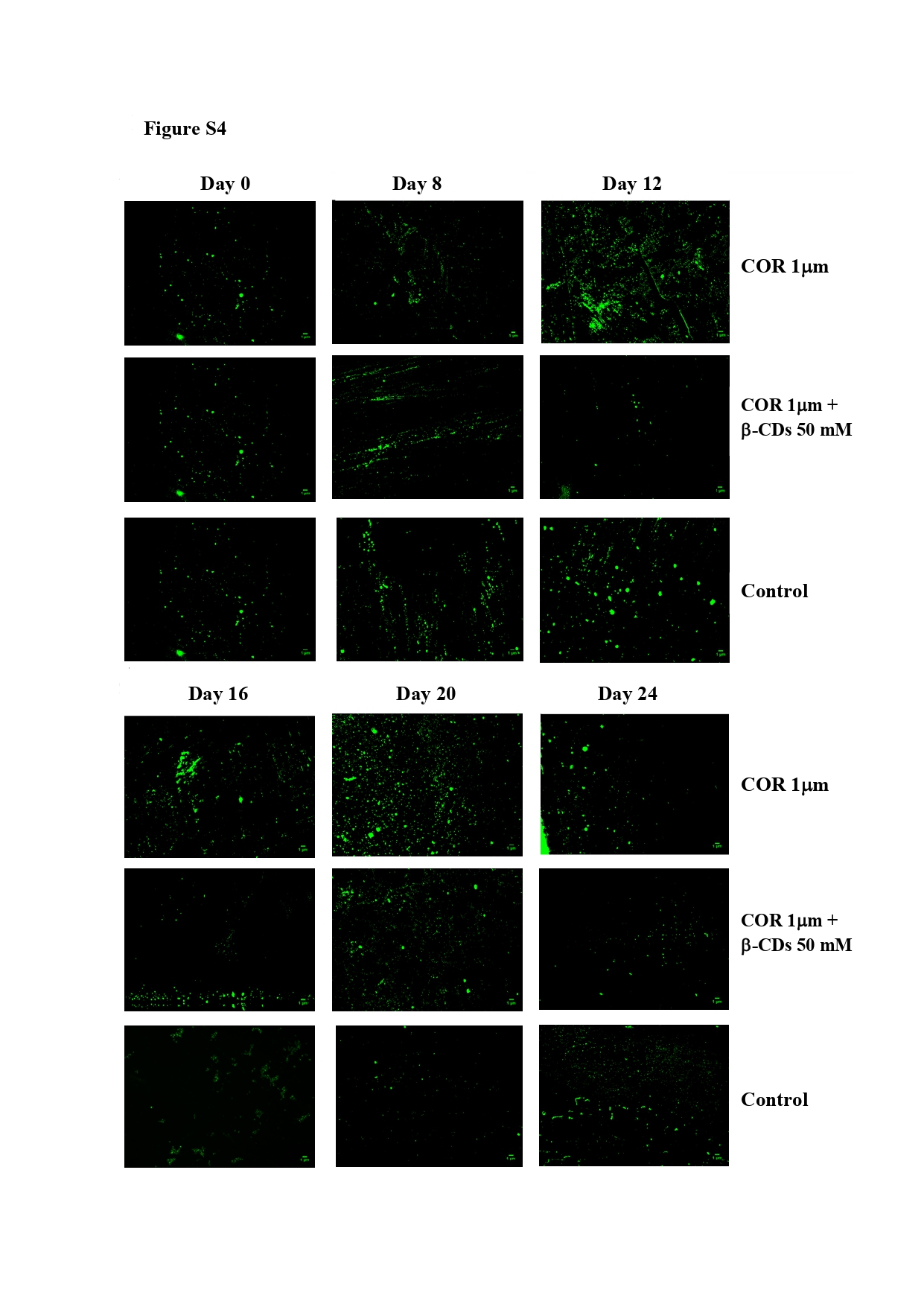

Supplement: Supplementary Figure 4 — Example of the size and abundancy of the isolated LDs from T. baccata cells cultured in Control, COR and CC conditions, throughout of the experiment by using a fluorescence microscope at 10X ocular and 10X objective. Size and abundance were calculated using ImageJ software and expressed as means ± SD (n = 10). [file Image_4.jpg]

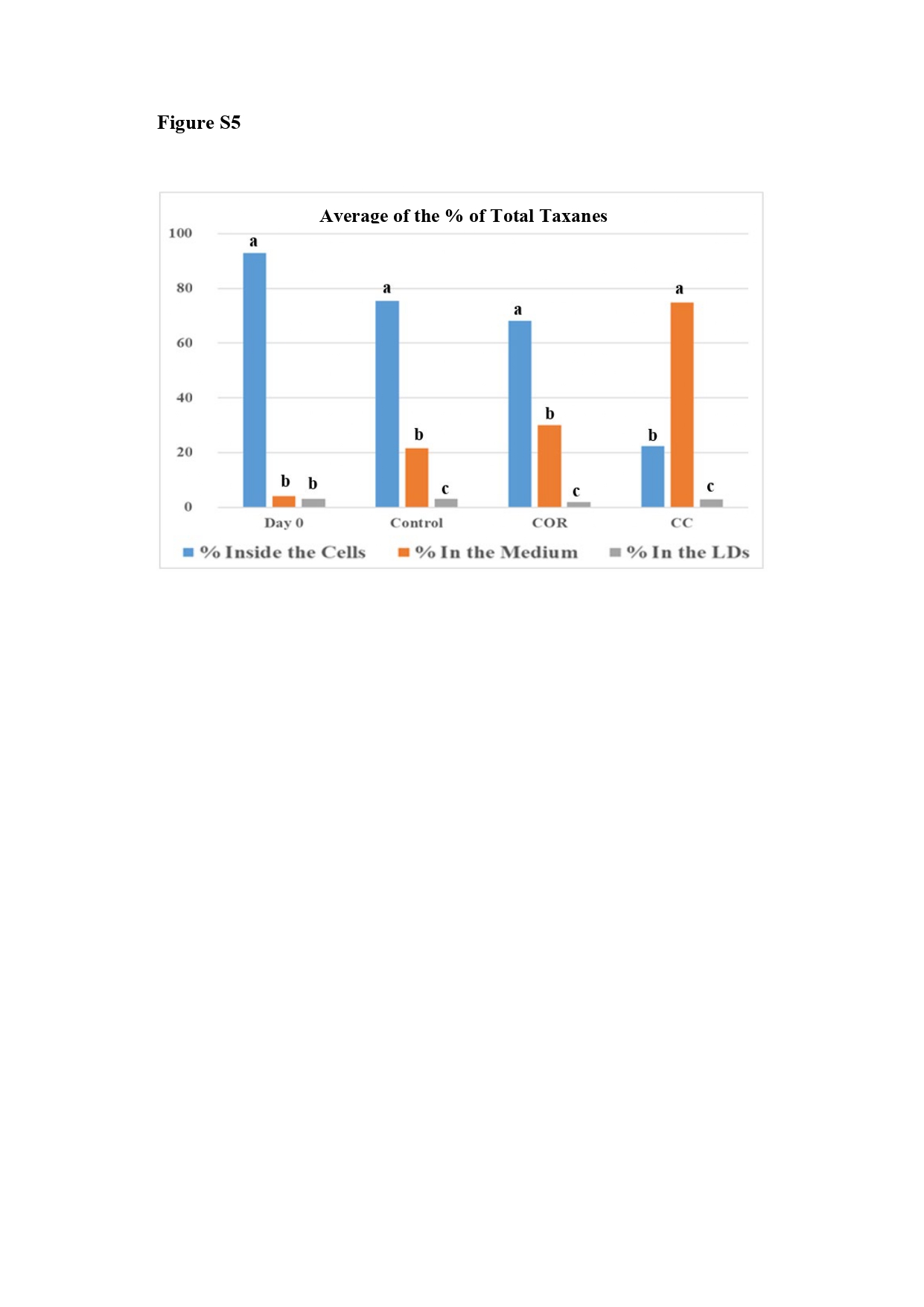

Supplement: Supplementary Figure 5 — Average sum of the different target taxanes found inside the cells, culture medium, and lipidic droplets (LDs) throughout the 24 days of the study. T. baccata cell cultures were maintained in control conditions or with the addition of 1 mM coronatine (COR) or 1 mM coronatine + 50 mM β-cyclodextrins (CC). Values presented are means ± SD (n = 3). Values followed by different letters are significantly different (P ≤ 0.05) according to Tukey’s honestly significant difference test. [file Image_5.jpg]

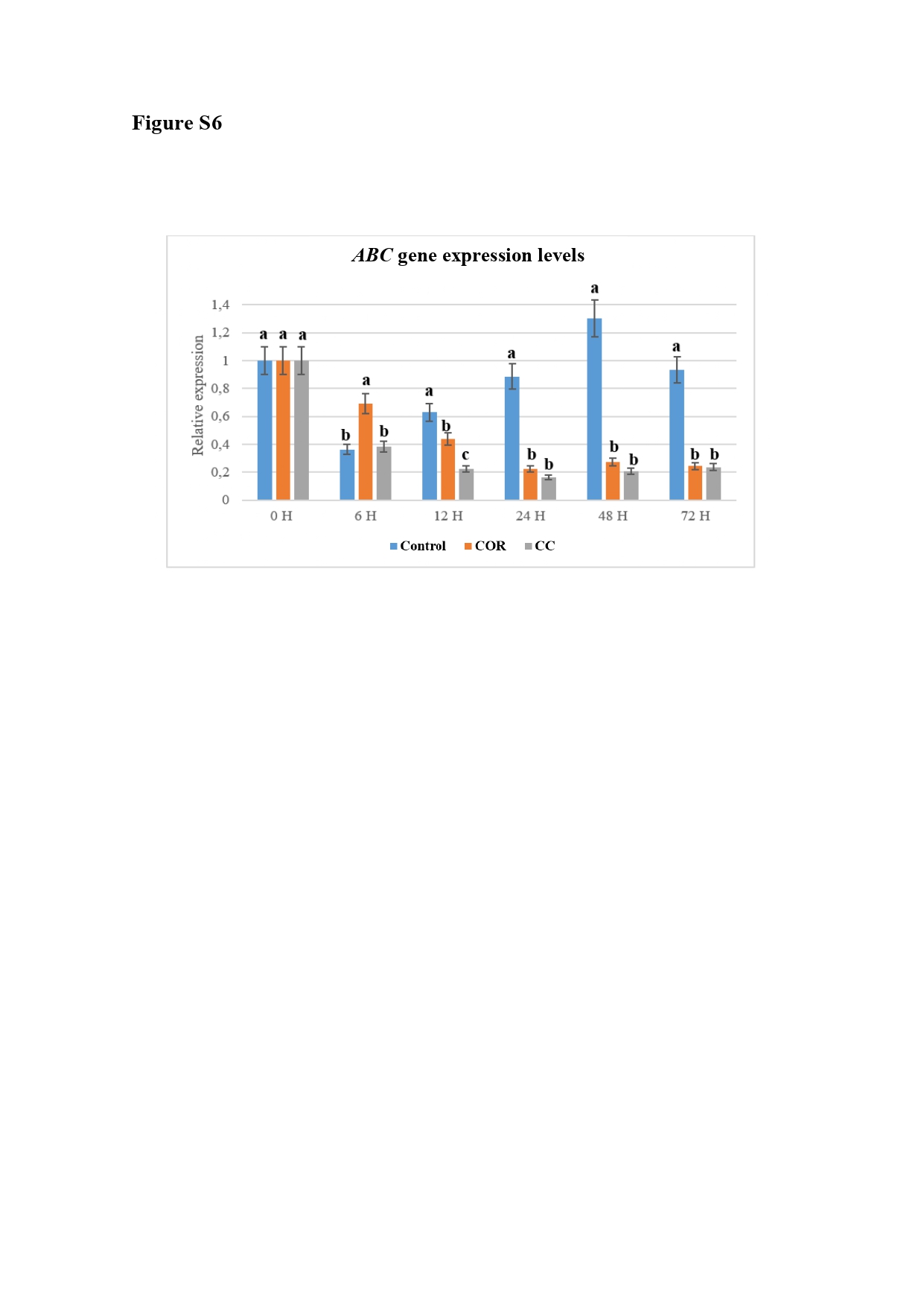

Supplement: Supplementary Figure 6 — Relative expression of the ABC gene in T. baccata cell cultures grown, for 72 h, in Control conditions and elicited with 1 mM coronatine (COR) or 1 mM coronatine + 50 mM methyl-β-cyclodextrins (CC). Values presented are means ± SD (n = 3). Values followed by different letters are significantly different (P ≤ 0.05) according to Tukey’s honestly significant difference test. [file Image_6.jpg]
