# Supplementary material for: Insights into the control of taxane metabolism: Molecular, cellular, and metabolic changes induced by elicitation in Taxus baccata cell suspensions
Source: Front Plant Sci. 2022 Jul 29;13:942433. doi: 10.3389/fpls.2022.942433 (PMC9372332; doi:10.3389/fpls.2022.942433)
Supplement: Supplementary Table 1 — Sequences of the primers used to amplify the genes by RT-qPCR. [file Table_1.pdf]

Supplementary Table 1.

Table S1. Sequences of the primers used to amplify the genes by RT-qPCR.

Gene	*Primer sequences*	*Amplicon size*	*Reference*
TXS	Forward: 5'-TTC GCA CGC ACG GAT ACG-3'<br>Reverse: 5'-TTC ACC ACG CTT CTC AAT TCG-3'	115 bp	Onrubia et al., 2010
BAPT	Forward: 5'-TAA GCA CTC TAC AAC AAC AGG-3'<br>Reverse: 5'-GCA TGA ACA TTA GTA TCT TGA TTC C-3'	111 bp	Onrubia et al., 2010
T7βOH	Forward: 5'-GGT CCG CCC AAA TTG CCA GAA-3'<br>Reverse: 5'-CCC TGC AGA GCC CAA AAA ACC-3'	110 bp	Onrubia et al., 2010
DBTNBT	Forward: 5'-CGG GGG GTT TGT TGT GGG ATT A-3'<br>Reverse: 5'-TTA GCC TCT CCC CTC GCC ATC T-3'	105 bp	Onrubia et al., 2011
ABC	Forward: 5'-AGC CTA TGC ATC CCT AGT GCA A-3'<br>Reverse: 5'-GTT GCC TGC CAG TGT TAT TT-3'	119 bp	Sabater-Jara et al., 2014
TBC41	Forward: 5'-CAA GAA GAA AGA GTC AGC AAA TGG-3'<br>Reverse: 5'-GGA ACG ACA TGA CAT TAT GAA TAG C-3'	91 bp	Sabater-Jara et al., 2014
